# The relationship between preoperative serum vitamin D deficiency and postoperative atrial fibrillation: A systematic review and meta-analysis

**DOI:** 10.34172/jcvtr.2021.25

**Published:** 2021-03-18

**Authors:** Mehran Rahimi, Mohammadreza Taban-Sadeghi, Leila Nikniaz, Fariba Pashazadeh

**Affiliations:** ^1^Student Research Committee, Cardiovascular Research Center, Tabriz University of Medical Sciences, Tabriz, Iran; ^2^Cardiovascular Research Center, Tabriz University of Medical Sciences, Tabriz, Iran; ^3^Tabriz Health Services Management Research Center, Health Management and Safety Promotion Research Institute, Tabriz University of Medical Sciences, Tabriz, Iran; ^4^Research Center for Evidence-based Medicine, Iranian EBM Centre: A Joanna Briggs Institute (JBI) Centre of Excellence, Faculty of Medicine, Tabriz University of Medical Sciences, Tabriz, Iran

**Keywords:** Vitamin D, Vitamin D Deficiency, Atrial Fibrillation, Postoperative Period, Cardiac Surgical Procedures

## Abstract

Postoperative atrial fibrillation (POAF) is the most common arrhythmia seen in the first days following cardiac surgeries. Recently, there is a growing discussion regarding the link between vitamin D deficiency and POAF development. This systematic review and meta-analysis of the observational studies aimed at evaluating the association between preoperative vitamin D deficiency and Postoperative atrial fibrillation. In this study, using PubMed, Scopus, Google Scholar, EMBASE, Web of Science, and Cochrane Libraries, we searched for records published before July 2020. Two reviewers screened for studies that examined the relationship between preoperative vitamin D levels and the generation of POAF. Data regarding study design, patient characteristics, definition of atrial fibrillation (AF), type of surgery, vitamin D levels, and measurement methods were extracted. Five studies were included in the meta-analysis. Our primary analysis showed a significant relationship between preoperative levels of vitamin D and POAF development (mean differences (MD) = -2.851, 95% confidence interval (CI) =-5.506 to -0.195; *P* value 0.035). Our meta-analysis suggested serum vitamin D deficiency is associated with an increased risk of POAF development. Further large scale interventional studies are needed to explore whether vitamin D supplementation will prevent POAF.

## Introduction

As the most common arrhythmia in the first days following cardiac surgeries,^1^ atrial fibrillation(AF) is seen in 0.51% of the worldwide population and has faced a 33% rise in its prevalence through recent two decades.^2^ This arrhythmia, increasing cerebral emboli up to 50%, is an important cause of morbidity and mortality in elder patients.^3^ Postoperative atrial fibrillation (POAF) is mostly seen in the first 4 days after surgery.^4^ Combined valve and coronary artery bypass surgery has the highest prevalence of POAF (60%-80%) among different types of cardiac surgery.^5^ Systemic and pericardial inflammation, tune changes in the autonomic nervous system and oxidative stress are examples of physiological disorders in cardiac surgeries thought to be possible stimulators for POAF.^6^ Furthermore, recently there is a growing discussion on the role of vitamin D in POAF development.

Vitamin D, an antioxidant agent, plays an important role in the renin-angiotensin-aldosterone system **(**RAAS), helping to maintain blood pressure in a range that can decrease atrial remodeling happening in atrial fibrillation. Vitamin D inhibits the RAAS system, has a reverse correlation with angiotensin ll and its deficiency can be damaging because of the RAAS negative feedback. ^7,8^ Moreover, high levels of C-reactive protein, which is associated with atrial fibrillation, can be seen in vitamin D deficiency. ^9,10^

There is some controversy on the link between vitamin D levels and the generation of atrial fibrillation. While some studies have shown a significant positive relationship,^11,12^ others have demonstrated no or negative relationship.^13-15^ To the best of our knowledge, there is no study focusing on the association between preoperative vitamin D deficiency and POAF generation. Therefore, we aimed to perform a systematic review and meta-analysis to assess this link.

## Methods

This study was performed according to the Preferred Reporting Items for Systematic Reviews and Meta-Analysis (PRISMA) guidelines. ^16^

### Eligibility criteria

The inclusion criteria for the studies were: (1) observational studies that had assessed the aforementioned link and (2) articles written in English. Case reports, review articles, conference abstracts, letters, comments, studies regarding surgeries other than cardiac surgeries, articles written in other languages, and studies that measured serum vitamin D after surgery were excluded. Preoperative AF was also considered an exclusion criterion in this study.

### Search strategy

Studies conducted were screened in accordance with the PRISMA flow diagram. A comprehensive literature search was performed using PubMed, Scopus, ProQuest, Google Scholar, EMBASE, Web of Science, and Cochrane Libraries up to June 2020. Search terms in PubMed included: “Vitamin D Deficiency”[Mesh], vitamin D[Title/Abstract], “Atrial Fibrillation”[Mesh], atrial fibrillation[Title/Abstract], “Postoperative Period”[Mesh], postoperat*[Title/Abstract], post-operat*[Title/Abstract], “Coronary Artery Bypass”[Mesh], surgery[Title/Abstract] and “Cardiac Surgical Procedures”[Mesh]. Unpublished or gray literature was identified by searching bibliographies from the retrieved studies manually.

Study selection and data extraction:

The search was performed by two independent investigators (M.R. and F.P.), then duplicate results were removed and studies were selected based on titles and abstracts. We used Endnote X6 for organizing and screening the titles and abstracts and removing the duplicates. Two independent reviewers (M.T and F.P) assessed the selected studies for inclusion eligibility based on the full text and data were extracted using a standardized Microsoft Excel form (Microsoft Corporation, Redmond, Washington). Information was collected regarding study type, number of the cases and controls, definition of AF, the method for measurement of vitamin D, the time of sampling for vitamin D, and type of surgery.

### Quality appraisal

The quality of the included studies was evaluated based on the Joanna Briggs Institute (JBI) Checklists for cohort studies.^17^ Quality assessment was completed by two authors (M.R. and M.G.) on separate occasions. Any disagreement during the process was resolved by consensus or consultation with a third reviewer (M.R.).

### Data synthesis and Analysis

The data were combined and expressed in mean and standard deviation (SD). One of the studies^18^ reported vitamin D levels in nmol/L. 2.496 was used as the conversion factor to convert nmol/l to ng/ml.^19^ We used formula by Hozo et al to estimate mean and SD for the data reported as median (min-max). ^20^

The meta-analysis was performed using Open Meta-Analyst® software (Brown University, Rhode Island, USA). The mean difference was calculated. The sensitivity analysis was done with leave-one-out meta-analysis to assess the stability of results and to verify that our findings were not driven by any single study.^21^ Heterogeneity was evaluated by I^2^ statistics. In case of an I^2^ greater than or equal to 50%, a significant heterogeneity of results was acknowledged. A *P* value of <0.05 was considered statistically significant. We performed the meta-analysis following the Cochrane Collaboration recommendations and reported the results according to the Preferred Reporting Items for Systematic Reviews and Meta-Analyses **(**PRISMA**)** statement. ^16,21^

## Results

**Figure 1:**
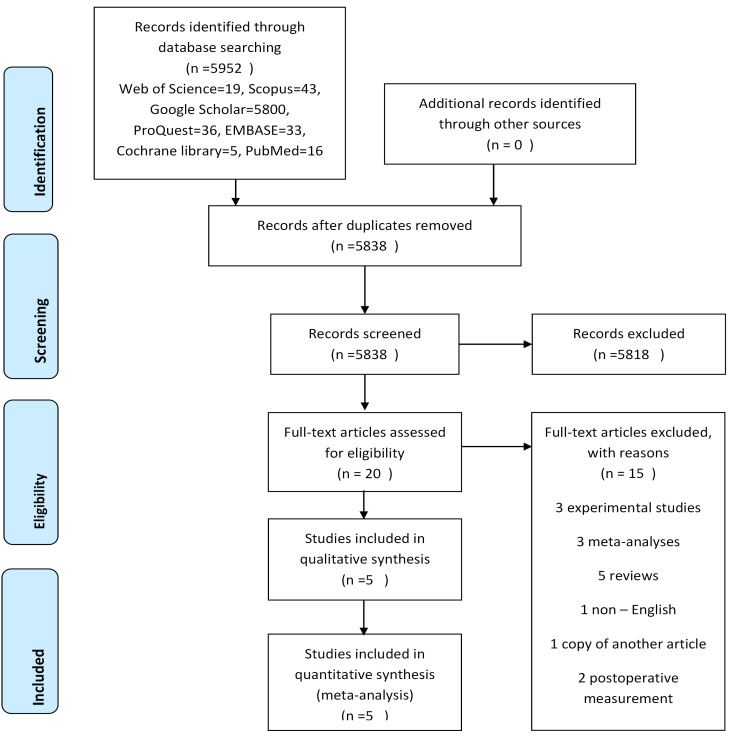
PRISMA flow diagram

The original literature search retrieved a total of 5952 results from the databases mentioned in the search strategy section. After the selection process, a total of 20 studies underwent full-text assessment. Subsequent to the exclusion of 15 papers, 5 ^18,22-25^ were included in the qualitative synthesis. The reasons for exclusion studies are shown in Figure 1.

### Study characteristics and quality

The characteristics of the included studies are demonstrated in Table 1. Five studies with 219 AF cases and 669 participants were included in this systematic review. Overall, these studies were published between 2016 and 2017. Among the included articles, four were nested-case-control (prospective case-control) studies and one was a retrospective cohort study. All of the studies examined the link between serum vitamin D levels measured before surgery and POAF in patients undergoing cardiac surgeries. All of the included studies were of high quality (Table 2). The shortest period of follow-up (48 hours postoperatively) for POAF was reported in Shadvar et al’s study. ^22^

**Table 1:** Study characteristics

**Author**	**Country**	**Population**	**POAF**	**SR**	**Mean age**		**MVD POAF**	**MVD SR**	**Definition of AF**	**Method for measurement of vitamin D**	**Type of study**	**Type of surgery**
					**POAF**	**SR**						
Skuladottir et al 2016	Iceland	118	66	52	70·5(med)	64(med)	19.862 ± 8.823	17.748 ± 9.534	Continuous electrocardiaographic monitoring	MS/MS Vitamin D Kit	Nested case-control	CABG and/or valvular surgery
Emren et al 2016	Turkey	283	72	211	70 ± 8.6	61 ± 9	15.6 ± 7.4	19.1 ± 9.1	ECG	Chemiluminescence immuno assay	Nested case-control	CABG
Shadvar et al 2016	Iran	50	25	25	69 ± 0.3	72 ± 0.8	27.4 ± 2.22	28.2 ± 1.18	Holter monitoring	Not mentioned	Nested case-control	CABG
Gode et al 2016	Turkey	90	15	75	59.1 ± 5.4	58.4 ± 9.1	9.0 ± 5.0	15.0 ± 8.4	Five-lead telemetry	Not metioned	Nested case-control	CABG
Cerit et al 2017	Cyprus	128	41	87	67.6 ± 8.6	63.9 ± 9.8	19.9 ± 6.1	26 ± 8.2	Daily electrocardiographic recordings	Chemiluminescence immunoassay	Retrospective cohort	CABG
total		669	219	450							

Abbreviations: MVD, mean vitamin D; POAF, postoperative atrial fibrillation; SR, sinus rhythm

**Table 2:** Quality assessment

**Article**	**Were the two groups similar and recruited from the same population?**	**Were the exposures measured similarly to assign people to both exposed and unexposed groups?**	** Was the exposure measured in a valid and reliable way?**	**Were confounding factors identified?**	** Were strategies to deal with confounding factors stated?**	**Were the groups/participants free of the outcome at the start of the study (or at the moment of exposure)?**	** Were the outcomes measured in a valid and reliable way?**	**Was thefollow up time reported and sufficient to belong enough for outcomes to occur?**	**Was follow up complete, and if not, were the reasons to loss to follow up described and explored?**	**Were strategies to address incomplete follow up utilized?**	**Was appropriate statistical analysis used?**	**TOTAL SCORE**
Skuladottir et al	YES	YES	YES	YES	YES	YES	YES	YES	YES	YES	YES	11
Emren et al	YES	YES	YES	YES	YES	YES	YES	YES	YES	YES	YES	11
Shadvar et al	YES	YES	UNCLEAR	YES	NO	YES	YES	YES	YES	YES	YES	9
Gode et al	YES	YES	UNCLEAR	YES	YES	YES	YES	YES	YES	YES	YES	10
Cerit et al	YES	YES	YES	YES	YES	YES	YES	YES	YES	YES	YES	11

### Risk of bias within studies

Two studies^22,24^ did not report the method for measuring vitamin D and strategies to deal with confounding factors were not mentioned in Shadvar et al’s study. ^22^

### Outcome

**Figure 2:**
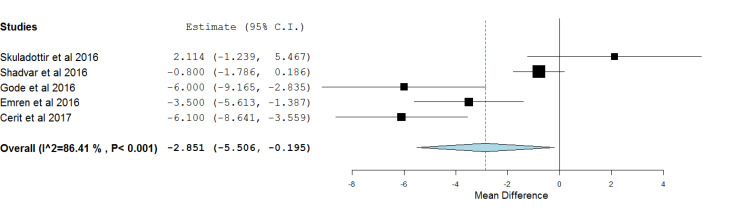
Forest plot

The meta-analysis was conducted based on preoperative levels of vitamin D. According to the identification of statistical heterogeneity (tau2:7.555, Q: 29.425, df (4), *P* < 0.001, I2: 86.406%) the continuous random-effects model was used. Our primary analysis showed a significant relationship between preoperative levels of vitamin D and POAF development (MD = -2.851, 95% CI= -5.506 to -0.195; *P* value 0.035). The forest plot is illustrated in Figure 2.

### Leave-one-out sensitivity analysis

**Figure 3:**
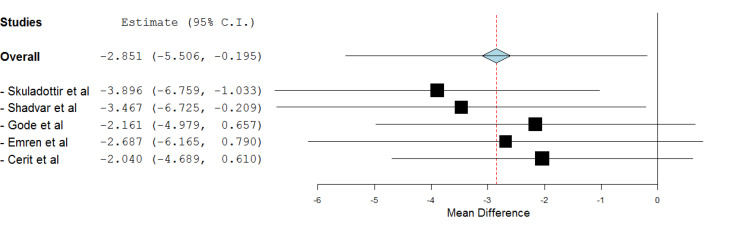
Leave-one-out meta-analysis forest plot

A leave-one-out meta-analysis was performed to evaluate the robustness of the results. No excessive influence was seen as the estimate of omitting an individual study, for all of the studies, did lie inside the 95% CI of the combined analysis. The forest plot is depicted in Figure 3. The study by Skuladottir et al reported contrasting results to other studies. The vitamin D levels in POAF group, surprisingly, were higher than its levels in sinus rhythm group.^18^

## Discussion

The present meta-analysis suggests that vitamin D deficiency, based on the preoperative levels, is significantly associated with POAF development (*P* < 0.05). To the best of our knowledge, this is the first study investigating the relationship between preoperative vitamin D deficiency and POAF.

One of the studies (Skuladottir et al) reported higher vitamin D levels in POAF group which was in contrast to the results of other articles. ^18^ All of the other studies stated that vitamin D levels were lower in the POAF group. This contradiction may be a result of the significant difference in terms of age between the POAF and control groups observed in Skuladottir et al’s study. In spite of the significant difference in respect to age in studies by Emren et al and Cerit et al, the vitamin D levels in those two studies were lower in POAF group.^23,25^ Only one of the multivariate regression models employed in these three articles resulted in a *P* value less than 0.05 for age: Skuladottir et al’s study. ^18^ According to the literature, as age advances, the burden of AF is increased and a sharp incline after age 65 years is seen. Thus, “age is the most important risk factor for AF”. ^26^ Based on the mentioned above, it can be deduced that age is a powerful confounding factor in these studies and matching of patients by age is essential.

There is some controversy regarding the relationship between vitamin D deficiency and atrial fibrillation. While a 2012 cohort consisting of 10 899 patients found that vitamin D deficiency is related to coronary artery disease, cardiomyopathy, diabetes, and atrial fibrillation,^11^ Skuladottir et al reported no significant relationship between vitamin D deficiency and POAF. The disagreement can also be seen in different meta-analyses conducted in this regard. Although two meta-analyses ^15,27^ did not found a significant link, a meta-analysis with more subjects found vitamin D deficiency to be associated with atrial fibrillation.^18^

Two systematic reviews and meta-analyses ^28,29^ were published addressing the same question. Neither of the studies did pay attention to the timing of vitamin D measurement. These studies combined data from both types of articles (preoperative and postoperative measurement of vitamin D). Several studies have stated that vitamin D levels drop significantly after surgery.^18,30-33^ Skuladottir et al reported a statistically significant difference between pre- and postoperative 25(OH)D2, 25(OH)D3, and total 25(OH) vitamin D levels. ^18^ The postoperative reduction in vitamin D values has been shown in different types of surgeries. Visser et al purposed that the reason for the postoperative drop in serum 25(OH)D3 concentrations is hemodilution. They reported a decrease in hematocrit levels after surgery which did not correlate with changes in serum 25(OH)D3 levels, but in their study both hematocrit and vitamin D levels returned to baseline. ^32^ Increased intracellular uptake of 25(OH)D3 after surgery vis-a-vis to a higher demand for tissue regeneration, an increased volume of distribution, catabolism, and clearance of vitamin D-related substances from the blood or increased urinary loss (as reduced serum vitamin D-binding protein and increased urinary DBP/creatinine ratio immediately after surgery was observed) are other possible pathophysiologies hypothesized by Binkley et al^34^ Considering that we decided to perform meta-analysis only based on studies which measured vitamin D levels preoperatively.

The pathophysiological mechanisms involved in the association between vitamin D deficiency and AF are as follow: activation of the renin-angiotensin-aldosterone system (RAAS),^8^ atrial electromechanical delay,^35^ decreased duration of action potentials in the left atrium^36^ and elevated C-reactive protein levels^9^.

One of the included studies ^22^ did not find a significant associotion between vitamin D deficiency and POAF development. This study was performed in Iran. Also, in a study conducted in Iceland measuring both pre- and postoperative levels, surprisingly, 25(OH)D2 levels were found to be higher in POAF group (independent predictor for POAF according to uni- and multivariate analysis) and there was no significant difference in levels of 25(OH)D3 and total 25(OH)D between two groups. Three studies (Gode et al Emren et al and Cerit et al) reported vitamin D as an independent predictor for POAF according to univariate analysis and except Cerit et al’s study, this link was also reported as assessed by multivariate analysis.^23-25^

Only in one study^18^ both coronary artery bypass grafting (CABG) and valvular surgeries were also included, but according to multivariable logistic regression analysis, the type of the surgery was not a risk factor for POAF. The rest of the studies included only CABG.

Firstly, all of the included studies were observational studies and the high heterogeneity observed in this study may be due to limitations of observational studies (as causation cannot be proven by these studies). Secondly, our study was also limited because of the small number of studies and unfortunately, the possibility of publication bias was not assessed. Thirdly, another limitation was because of the significant heterogeneity seen in this study. The study conducted by Skuladottir et al was found to be causing heterogeneity, as the results were completely in contrast with the rest of the studies. Fourthly, only studies in the English language were included, thus, some useful sources of evidence may have been missed. We believe that this meta-analysis, despite these limitations, does provide valid insights into the role of vitamin D deficiency in the generation of POAF.

## Conclusion

This meta-analysis suggested a positive association between vitamin D deficiency and POAF development. Further large scale studies are needed to determine whether there is a direct causal relationship between vitamin D deficiency and postoperative atrial fibrillation. Well-designed and well-executed randomized clinical trials evaluating the effect of vitamin D supplementation on the prevention of POAF would resolve this debate.
